# Key features of an innovative sub-acute residential service for young people experiencing mental ill health

**DOI:** 10.1186/s12888-019-2303-4

**Published:** 2019-10-23

**Authors:** Rachael Green, Penelope Fay Mitchell, Kira Lee, Ella Svensson, Jia-Wern Toh, Carolyn Barentsen, Michala Copeland, J. Richard Newton, Kari Christine Hawke, Lisa Brophy

**Affiliations:** 1 Centre for Mental Health, Melbourne School of Population and Global Health, The University of Melbourne, 207 Bouverie Street, Carlton, Victoria 3053 Australia; 2 Orygen, the National Centre of Excellence in Youth Mental Health, 35 Poplar Roadd, Parkville, Victoria 3052 Australia; 3 Peninsula Health, 2 Hastings Road, Frankston, Victoria 3199 Australia; 4 Mind Australia, 86-92 Mount Street, Heidelberg, Victoria 3084 Australia; 5 School of Allied Health, Human Services and Sport, La Trobe University, Bundoora, Victoria Australia

**Keywords:** Young people, Mental illness, Recovery, Community mental health, Residential care

## Abstract

**Background:**

Numerous studies across international settings have highlighted a need to improve the appropriateness and continuity of services for young people experiencing mental ill health. This paper examines key features of a sub-acute youth mental health residential service model, Youth Prevention and Recovery Care (Y-PARC) service. Y-PARC provides up to 4 weeks care to 16 to 25 year-olds at risk of hospitalisation and to those transitioning out of hospital inpatient units. The research was conducted at one of three Y-PARCs located in Victoria, Australia.

**Methods:**

This paper presents findings from analysis of two data sources collected during evaluation of a Y-PARC service in 2015–17. Routinely collected administrative data of Y-PARC residents (*n* = 288) were analysed and semi-structured interviews were conducted with 38 participants: a) former residents (*n* = 14); b) family members of group a) (*n* = 5); key stakeholders (*n* = 9); and, Y-PARC staff (*n* = 10 respondents in 3 group interviews). Analysis of the qualitative data was thematic and structured by the interview guide, which covered the key service aims.

**Results:**

Consistent with the aims of the service, respondents described practice at Y-PARC that aligns with recovery-oriented care. Key features emphasised were: a safe and welcoming environment for residents and families; provision of person-centred care; promotion of autonomy and self-help; informal interactions with staff allowing for formation of naturalistic relationships; time spent with other young people with similar experiences; and, assurance upon exit that the ‘door is always open.’

High levels of satisfaction were reported. Outcomes described included: improved resilience; better understanding of mental health; the importance of seeking help; and, stronger connections to therapeutic services. Longer and multiple stays were associated with progressive and sustained change. Family members and stakeholders widely reported that the service fills a gap between community services and acute inpatient mental health hospital wards.

Some challenging areas of practice identified included: integration of evidence-based psychosocial interventions; provision of care within a model that blends clinical and psychosocial support services; and, negotiation of family-inclusive practice.

**Conclusions:**

The Y-PARC service model shows promise with young people experiencing mental ill health, particularly in improving the range and availability of options across a spectrum of need.

## Background

Youth mental ill health is a significant issue worldwide, affecting individuals, families and communities. The ‘global burden’ of mental and substance use disorders is estimated to have increased by 37.6% between 1990 and 2010 [1], and is one of the main contributors to disability in young people [2–4]. Researchers estimate that a quarter of mental disorders emerge before the age of 12 years and about three-quarters before the age of 25 [5]. The impact on social, educational, vocational and developmental milestones can be severe [6] and often persist into adulthood.

Despite this high level of need, engagement with mental health services by young people aged 12–25 years is the poorest of all age groups [5]. Experts have advocated for systemic change to improve the care provided to children and young people and boost the inclusion of families/significant others [7–9]. In particular, there has been emphasis on early identification and engagement of children and young people experiencing mental ill health, increased availability of specialist mental health care and improved coordination of care [3, 7, 9]. A key issue identified in numerous studies is that the difficulties involved in transitioning between child and adult health care systems often result in disengagement [10–13].

In Australia, there has been a longstanding recognition of the importance of improving transitions and continuity of mental health care [14, 15]. Nevertheless, fragmentation and lack of coordination of mental health care are ongoing service system issues [16]. Researchers have argued that ‘sub-acute’ residential programs are needed to fill an important gap in the system [17, 18], providing more intensive support than the community (‘step-up’ care) and assisting with transitions back into the community following hospital admission (‘step-down’) [19].

Provision of sub-acute residential services for adults has become increasingly common in Australia [17, 20, 21]. Recovery-oriented models of care are embedded into many of these [19, 22–24]. Recovery-oriented mental health services are generally understood to feature the following: consideration of individual needs and wants; empowerment and promotion of self-care; embracing individual strengths and resilience; acknowledgment that the path to recovery is unique and varied; enabling those affected to benefit from one another; and promoting greater acceptance of people with mental health issues in the community [22, 23, 25–27]. An evaluation of an adult step-up step-down recovery-oriented service in Australia found that clients had significant improvement in symptoms and levels of impairment at exit [21]. Clients placed value on the following recovery-focused features: support to reduce symptoms, the opportunity to practice development of social relationships, and the opportunity to develop self-care skills [21]. However, researchers have noted that therapeutic models within sub-acute residential mental health care services are not well described or evaluated [24, 28]. This is more so the case for youth-focused sub-acute residential services, which are a more recent innovation [28, 29].

The relatively modest amount of recent literature investigating models of therapeutic care for young people in residential settings is framed by an overall decline in use of residential care settings such as ‘group homes’ with vulnerable children and young people [30, 31] and shift toward ‘least restrictive’ community-based mental health care [32]. Despite these shifts, the potential efficacy of short-term, structured, needs-based, therapeutic residential care has gained recognition [32]. For example, a U.S. study [33] reported that intensive short-term residential treatment can effectively treat adolescents with severe psychiatric problems, finding that adolescents had sustained improvements in the year following discharge. Evaluation of a secure, residential treatment program in the U.S., focusing on “ecological outcomes” and utilising “a systems approach to emotional and behavioral problems”, found that, for those young people with more severe and persistent mental health issues, the program functioned to “deflect” the need for more intensive or restrictive intervention ( [34], p499).

The general literature on effective mental health service provision to young people shows provision of holistic care tailored to individual needs, flexible delivery [15] and therapeutic alliance between the practitioner and young person [35–37] are critical. Carer/family involvement in residential care settings is acknowledged as being important where possible [23, 38], with psychotherapeutic approaches involving families being supported by a strong evidence base [39]. Provision of developmentally appropriate care is emphasised. For example, one evaluation found that young people who are grouped in the 16–25 year age range have different developmental needs that should be accounted for in practice [28]. Three priority areas for young people in residential services were highlighted in the same study: employment and education, physical health and housing needs.

Consistently, the provision of ‘youth friendly’ care has been shown to be critical to improving engagement and retention of young people in services [40–42]. A scoping review identified that principles of ‘youth friendly’ care must be embedded across the following characteristics: organisation and policy; environment; service provider, and treatment/service [43]. Emphasis was placed on the importance of young people having a voice and being engaged in planning, development, implementation and service delivery [43]. A qualitative study seeking young people’s perspectives on psychiatric inpatient care found that the support of peers was frequently regarded as one of the most helpful aspects of hospitalisation, as were group therapy and the opportunity for ‘time out’ [44]. Participants also particularly valued interpersonal interactions with staff and the opportunity to learn coping strategies. Nevertheless, studies drawing on experiences of young consumers in residential therapeutic settings are uncommon.

The combined evidence from research with young people in both residential and non-residential settings indicates that there is a strong overlap between practice that is youth focused and features of recovery-oriented care. Examples include emphasis on individualised care, the instrumental nature of social support from family and peers and the relational aspects of treatment, including interaction with staff [45–47]. However, there is a gap in research that explore of features of recovery-oriented practice within the youth-focused community-based residential care service context. This is significant given the potential of sub-acute models of care in creating a more seamless service system and use of recovery-oriented principles in supporting therapeutic pathways to wellness for young people experiencing mental distress.

This paper reports the findings from an independent evaluation of one Y-PARC service located in an outer metropolitan area of Melbourne conducted in 2015–2017. Prevention and Recovery Care (PARC) services have sought to fill a gap in the continuum of mental health treatment and care by enhancing consumer access and options required for their individual needs [48]. These services initially targeted adults (over 18 years), providing short term (up to 28 days) mental health residential support and have been described elsewhere [20, 24]. In Victoria, this service has now been extended through the more recent establishment of Youth PARCs (Y-PARCs). Y-PARCs are tailored to the needs of young people between the ages of 16 and 25 [49]. Y-PARCs are explicitly recovery-oriented and operate using a blended service model, which involves a partnership between Mental Health Community Support Services (MHCSS) and public area mental health services, commonly referred to as clinical services. The short length of stay and therapeutic focus on mental health goals using a recovery framework are distinguishing features of the Y-PARC, with existing youth residential models in Victoria being for longer term stays (e.g., 1–2 years) and primarily focused on housing needs or education [50]. At the time of writing there were 21 adult PARCs located in Victoria but there were only three Y-PARCs.

In addition to gaining a better understanding of the reach of the service and characteristics of residents, the evaluation sought to identify ways to: 1) improve provision of therapeutic, recovery-oriented treatment and care; 2) strengthen family engagement and involvement; 3) understand and evidence the impact of Y-PARC on residents’ mental health status and on reducing demand at other acute medical and other mental health services; and 4) improve the service’s partnering arrangement. Analysis presented in this paper outlines identified key strengths of the service model as well as limitations and challenges experienced in service delivery that may be considered in development and improvement of youth mental health services both in Australia and elsewhere. Furthermore, this study contributes to a scant body of research in this area that draws on the perspectives of consumers and their family members/carers.

## Methods

This study draws on data collected during a mixed methods evaluation of the Y-PARC service, 2015–17 (Mitchell, Green et al. 2018).^1^ The evaluation aims were informed by a service ‘logic model’ describing the aims of the service. Development of key research questions was a collaborative process overseen by a Governance Group representing the four agencies involved in the evaluation (the clinical service, two MHCSS and the university-based research team). The Governance Group also oversaw the data collection process, analysis and development of recommendations.

While this study utilised data ‘about’ young people who accessed the service (e.g., analysis of the service’s database), particular value was placed on the experiences of service users. The perspectives of Y-PARC users and their carers were gained in in-depth interviews and analysed alongside information given by staff and stakeholders to enrich understanding of the service model, practice elements and impact. Interpreted within a hierarchy of user participation [51], the evaluation approach was informed by an understanding of the value of seeking contribution from young service users in active, empowering and capacity-building roles [52] and disruption of hierarchical relationships characterising research interviews. Therefore, young people who have been consumers of mental health services were engaged as co-researchers and contributed to the design of research questions, co-facilitation of qualitative interviews, data analysis, development of recommendations, writing of an evaluation report and this manuscript.^2^ The involvement of youth co-researchers in a genuine collegiate relationship has been ensured to avoid ‘tokenism’ [53]. The findings reported here follow the analysis of quantitative and qualitative data sources, described below.

### Resident characteristics

Secondary analysis of resident characteristics and outcomes data was conducted using data routinely collected and recorded on the mental health database and medical client information management systems. These de-identified and aggregated data describe age, gender, employment/education status, diagnosis grouping/s of residents who accessed the service across 3 years, 2012–15. Data were collated and basic descriptive analysis was conducted (involving calculation of percentages and proportions). Analysis contributes to understanding of the user group, patterns of access and reach of the service (presented under the “Characteristics of Y-PARC residents” heading below).

### Semi-structured interviews

Qualitative interview data were collected from four groups:

- Young people who have had an admission at the service (*n* = 14).
- Carers nominated by group a) (in this case all family members) (*n* = 5).
- Service providers who interact and liaise with the Y-PARC (key stakeholders) (*n* = 9).
- Y-PARC staff (3 group interviews conducted with *n* = 10 respondents).

### Procedure

A list of young people (over 18 years) who were residents of Y-PARC in the calendar years of 2014–16 was compiled. All young people on this list had been previously assessed by a psychiatrist on admission for ability to give informed consent. A letter was sent to a random sample of 150 young people from this sampling pool inviting them to contact the research team via telephone or email to volunteer their participation. The young people who were interviewed were also asked to nominate a family member or carer and these individuals were then invited to participate via a letter. Interviews were conducted face-to-face by a member of the research team and co-facilitated by youth researchers where possible. The average length of interviews was one hour. Young people and family member respondents were each reimbursed with a $40 shopping voucher for their contribution.

Service providers who interact and liaise with the Y-PARC were identified by the Governance Group. This list was supplemented by a local area scan conducted by the research team and a snowball method, which involved contacting stakeholders mentioned by interviewees via telephone or email. Individual interviews were conducted face-to-face or by telephone. Staff members with roles in managing the Y-PARC and delivering services in 2016 were invited to participate in one of two semi-structured group interviews.

Consistent with a constructivist approach [54] in-depth interviews were used to explore the subjective lived experience of four groups. All interviews were semi-structured using an interview guide that was tailored to each group as appropriate with a general focus across each on: exploring adherence of the service to its general aims, impacts (both in relation to individual outcomes and contribution to the system, depending on the perspective of the respondent), and identification of strengths and weaknesses of the service. Informed written consent was obtained and interviews were digitally voice-recorded.

All interviews were transcribed verbatim and personally identifying information was deleted from transcripts. Content analysis of the transcripts was undertaken, with this technique being “a careful, detailed, systematic examination and interpretation of a particular body of material in an effort to identify patterns, themes, assumptions, and meanings” ( [55], p183). Computer software NVivo version 11 (QSR International) was used to assist with the analysis, which involved ‘coding’ and interpreting of data [56]. The pragmatic aims of the evaluation study guided deductive or ‘a priori’ reasoning [57] whereby the questions and prompts in each semi-structured interview guide were used to develop a categorical scheme. Informed by a grounded theory approach, further sub-themes or ‘lower level codes’ were then identified inductively (derived from the data rather than from pre-established categories) [55] within each theme as the diversity of answers to the questions and prompts were discerned. Data were analysed separately for each respondent group. Because the higher-level themes were derived from the semi-structured interview schedule, similarities and differences between respondent groups on these themes could be discerned. Validation of the interpretive analysis was pursued in an iterative or ‘cyclical’ [58] process of seeking feedback from research team members as various drafts of the analysis were prepared. The initial draft was highly comprehensive and detailed, with subsequent drafts seeking to refine themes while reducing detail and length. The research team members included Y-PARC managers, youth co-researchers and academics. From these varied perspectives the research team discussed and edited numerous drafts in pursuit of a final agreed version.

Ethics approval for the analysis and reporting of the administrative data and the qualitative interview component of the research was gained through the Peninsula Health Human Research Ethics Committee (QA/15/PH/18 and LRR/16/PH/3). Reporting in this manuscript adheres to the Consolidated Criteria for Reporting Qualitative Research (COREQ) guidelines [59].

## Results

### Characteristics of Y-PARC residents

A total of 288 young people were residents at the Y-PARC in the three-year evaluation period (see Table 1). Numbers of young people accessing the service increased over this time, which was anticipated as the service opened in May 2012. Young people ranged in age from 16 to 25 years. The proportion of 16–17 year-olds increased from 13.75% in 2012–13 to 19.66% in 2014–15 and the proportion of 24–25 year-olds decreased from 15 to 12.82% over the same time. Females represented 60.8% of all residents over the 3 years.

**Table 1:** Y-PARC resident characteristics, episodes and average length of stay 2012–13 to 2014–15

Characteristic	Count/proportion
Resident numbers	*N* = 288
	2012–13, *n* = 80
	2013–14, *n* = 91
	2014–15, *n* = 117
Gender of residents count (proportion females to males)	Male *n* = 93; Female *n* = 194
	2.08
Episodes	370
Episodes to ratio of residents	1.28
Average length of stay in days	2012–13 = 19.7
	2013–14 = 19.1
	2014–15 = 20

Most young people (78%) who were admitted to Y-PARC during this period were admitted for the first time. Average length of stay was similar across each year, at 19.7 days in 2012–13, 19.1 in 2013–14 and 20.0 in 2014–15 (Table 1).

At Y-PARC a mental health diagnosis is made based on an initial assessment with reference to previous diagnoses with any discrepancies discussed in clinical review team meetings. Because specific diagnoses are often difficult to discern, and mixed presentations are the norm, broad diagnostic types are used to categorise the main presenting mental health issues (see Table 2). The most common diagnostic category among residents referred to emotional dysregulation such as borderline personality disorder.

**Table 2:** Primary diagnostic categories for Y-PARC residents 2014–15

Primary diagnostic category	%
Emotional dysregulation, personality disorder	38.4
Depressive episode (excl. Postnatal period)	22.4
Mixed anxiety and depressive disorder	8.7
Unspecified nonorganic psychosis	7.2
Adjustment disorders	4.3

### Characteristics of interview respondents

Characteristics of young people who participated in interviews are summarised in Table 3 below.

**Table 3:** Characteristics of Y-PARC residents

Gender	4 males, 10 females
Average age at interview	22.5 years (range 18–26)
Average age at last admission	20.5 years (range 16–24)
Number of admissions	1 stay, *n* = 8 2 stays, *n* = 3 3 stays, *n* = 1 4 or more stays, *n* = 2

All family member/carer respondents were mothers of young people who were interviewed/former Y-PARC residents (hence the sample is referred to as ‘family’ throughout). Three respondents spoke about how the young person had more than one visit to Y-PARC. One respondent had two children who had resided at Y-PARC (though at different times). The nine stakeholder respondents worked in six different not for profit health and community service and government agencies located in Victoria – with three respondents working in three different areas of one service and two respondents being from a government welfare agency. All stakeholders worked as team leaders or managers, with some also involved in direct service provision to young people. Ten of the pool of 17 staff who worked at the Y-PARC during the study period participated in group interviews. Respondents were comprised of three clinical staff members and seven MHCSS staff from across the three partner organisations.

### Perspectives of respondents on the Y-PARC service model and impacts

The five overarching themes that were explored in the semi-structured interview schedule and which guided the higher-level coding of the four data sets were:

1. Recovery-oriented practice.
2. Therapeutic environment.
3. Family engagement and involvement.
4. The partnering arrangement.
5. Impacts on mental health and wellbeing.

Sub-themes were identified under each heading and illustrative quotations are provided within sub-sections.

#### Recovery-oriented practice

Though the language “recovery-oriented” was not used by young people and family members, these respondents described features of Y-PARC that were consistent with both recovery concepts and with staff and stakeholder accounts of recovery-oriented practice. Four features were emphasised among the four groups.

##### Person-centred approach

Staff spoke about using a person-centred approach and how this differs from the more standardised ways of working in other services. Mirroring this, young people and families discussed how staff got to know young people and treated them like a “person”, and that this was quite different to their experience in hospital-based inpatients units.

*I mean they were doing the best job they could (at hospital mental health unit), but there wasn’t a lot of personal – there was no sort of personal – yeah, she was just another patient I suppose. Whereas at Y-PARC, you’re, you know, definitely a person, they know your whole background, they know your story. It obviously gets passed onto all the workers and they’re actually working to, you know, create – to help you progress. Whereas I think the hospital obviously are trying to just monitor your medication and you’re just another – sadly, just another … a number really. (Family #4)*

##### Promoting autonomy and self-help

Family members and staff spoke about how the service empowers young people and helps to build their emotional resilience and life skills by promoting autonomy through time spent away from family. All respondents spoke about young people’s improved help seeking skills and increased connection to helping professionals through staff referrals.

*We don’t do it for the client, we … will assist them and support them but we don’t actually do it. So that they get the idea that they’re in charge of their recovery so … they know that they have to go to appointments themselves. We don’t take them, but we give them as much information as possible about all the services that are out in the community. (Staff)*

Recovery plans were developed for all young people however young people reported that they would have appreciated more follow up regarding implemtation and progress.

##### Normalising experiences of mental health issues

Respondents spoke about how residents benefited from living together, partly due to the insight it gave them into the struggles of others with mental health issues and realising that they were not alone in their experience. Young people reported making friends at the service and family members reported that the young person was less socially isolated because of their stay/s.

*I think it showed her that she’s not the only one in the world that suffers like that. I think it showed her that there are ways of coping with it, and there are ways of working through it. (Family #3)*

##### Benefits associated with multiple stays

Respondents from each group reported that young people benefited from having multiple stays at Y-PARC, making progressive steps towards recovery with each visit. Initial stays were often focused on learning about mental health issues, building trust with the staff, and developing insight into their individual mental health needs. Developing this level of ‘readiness’ was understood as necessary before young people could engage with information about what kinds of treatments and support are available and begin to explore these options. Respondents reported that young people addressed their presenting problems more quickly with each stay and were reassured knowing that the ‘door is always open’.

#### Therapeutic environment

Four sub-themes were prominent in respondents’ discussion of the therapeutic environment at Y-PARC.

##### Safe and comfortable

With managing self-harm and suicide risk being highly prominent concerns, coming into a safe environment was critical for both young people and family respondents. Feeling “comfortable” was also important – and this most often referred to emotional state. Young people and family members spoke about how staff made them feel comfortable because they were friendly, welcoming and open.

*But seriously, I felt really comfortable with the environment there. I think I felt more comfortable with that than in my own bloody home (laughs) because you know – you just did. (Young person, Male #2)*

The elements of safety and comfort appeared to be building blocks in setting up a therapeutic environment.

##### Relationship-based approach

Young people and family members spoke about how, although professional boundaries were maintained, young people experienced and benefited from having personal interaction with staff facilitated through time spent together in informal environments. Many young people described experiencing a sense of friendship with select staff, having had the opportunity to get to know multiple staff members.

*But I think sometimes, when it comes to needing support, you kind of - not so much need a - need a friend as such, but just the relationship that you form is a little bit different than just to like a clinical kind of … (Young person, female #10)*

##### Holistic and developmentally appropriate

Numerous examples illustrating a holistic approach to practice were mentioned. Our data suggest that, for young people in a residential setting, a holistic approach to care includes environmental features and interventions that facilitate movement through developmental challenges. Opportunities to learn and practice skills of independent living were particularly valued such as establishing routines around sleep, exercise and eating well. Other issues included accommodation needs and working with family. Several family members reported that their child benefited from the opportunity to experience some independence.

*I quite liked that she was able to go on her own and not have my influence in any way so that she could find her own way […*] *I now understand that that’s what she needed was to be away from me and be in an environment nothing like her home and, you know, sink or swim. (Family, #2)*

Group living also brought some challenges, with young people appreciating fair and consistent enforcement of rules, and opportunities to learn from their mistakes without being excluded by the service.

##### Use of evidence-based psychosocial interventions

Staff emphasised the importance of establishing “the basics” of supporting young people during their limited time at the service. This related to relationship building, helping the young person feel safe and establishing day to day routines. Staff mentioned using techniques such as mindfulness and problem-solving strategies to assist with young people’s identified goals (e.g. managing anxiety). They also reported that they administered components of more structured, evidence-based psychosocial interventions such as cognitive behavioural therapy (CBT) and dialectical behavioural therapy (DBT) when working with young people who were further advanced in their recovery journey (i.e. during subsequent visits so the Y-PARC). However, due to time constraints, they did not implement full programs.

Some young people spoke about working on specific issues such as problem solving and sleep; however, many were not able to recall receiving any structured psychosocial interventions. Young people and family members reported that they would have benefited from doing so. Five young people specifically suggested a need for more structured group sessions focusing on skill building in line with recovery goals. Young people and family members both reported that the service would be enhanced by access to a clinical psychologist.

*Well, I think as great as the staff are with, you know, trying to teach coping strategies and all that sort of stuff, their knowledge only goes so far as their training has only gone so far … Whereas a psychologist has a much more in-depth knowledge into each sort of therapy … [Young person, Female #7]*

This was an area of tension. Respondents also spoke about how many young people were not able to fully engage in therapeutic activities during their stay due to the acute nature of their mental health state, and benefited from receiving assistance with “the basics” as well as from the informal, relationship-based interaction styles of staff. As mentioned above, multiple stays enabled young people to develop readiness for focused psychological strategies at their own pace.

#### Family engagement and involvement

Three main sub-themes emerged in relation to family engagement and involvement.

##### Family friendliness

Young people and family members consistently reported that the Y-PARC was welcoming and comfortable for visitors. The physical layout of the facility (having both shared and private spaces) was understood to be conducive to promoting family involvement as was the programming of a “community dinner” once a week (where visitors were invited by residents).

*It’s just a very welcoming, warm place I think, that you can walk in and you don’t feel as though you’re in a, you know, mental health place at all. It’s, it’s very homely, very comfortable. (Family #3)*

##### Availability of information for family

Even though most family members were satisfied with the information they received about the service at intake, they reported that they wanted information prior to intake so that they could assist the young person in making an informed decision about whether to enter the service.

*Well, she [daughter] came back from seeing her psychiatrist and had talked about Y-PARC and I was a bit like, well, what is it? And, you know, I tried to even find out, you know about it and really kind of couldn’t. [Then] she came and said, “Look, actually I’m deciding that I want to do this, I’m not really - I’m not coping outside.” (Family #5)*

##### Family involvement during residence

Young people mentioned that Y-PARC staff respected their wishes when they did/did not want family involved and described how staff helped to set up and facilitate family meetings when there was tension within families. Staff spoke about doing their best to work with families in a dynamic way, yet all experienced challenges and tensions negotiating an appropriate level of family involvement. Two of five family members gave examples of times where they felt they were not consulted or valued by the service, specifically in relation to prescribing decisions. Respondents valued the presence of a specialist Family Engagement Worker at the Y-PARC.

*Sometimes the family want to be involved a bit and the young person doesn’t want them involved as much as they want to be involved so we need to try to work with that tension as well and try to work with both as much as we can but also again with policies and procedures the law, the Act, you know. (Staff member)*

#### The partnering arrangement

The partnership between the clinical and the mental health community support services was spoken about by staff and stakeholders as a strength of the service and a challenge to implement. Staff spoke about how they had been able to learn from the staff from other agencies with different backgrounds and this has provided a stronger base for the holistic model of care provided to young people.

*The beauty and the challenge and complexity is the partnership arrangement between three organisations. (Staff member)*

Young people and family members spoke about problems that appear to have manifested from challenges in the integration of different practitioners in the service. For example, family members reported being confused about the roles of the various members of staff they interacted with in the service.

Many family and staff were critical of the limited availability and lack of continuity of the psychiatric care at the Y-PARC. Young people and family members spoke about how consulting with multiple psychiatrists or psychiatric registrars and not having continuity in this contact was detrimental to their care.

*I wouldn’t trust … say if I saw you … once a week and then someone else came in the next week and told me, “[W]hat’s happening with your medicine?” and I had to explain my story again and then that’s traumatic as it is and then you come in the next time and then someone else comes in, like I’m not going to trust any of you [laughs] like I’m not going to give you anything. So, it’s … not very comfortable, I suppose, for it to go that way. (Young person, Female #1]*

There was a strong preference among those who were engaged with a private practitioner external to the service to continue that arrangement.

#### Impacts on mental health and wellbeing

The majority of young people reported that they discerned improvements in their mental health that they could attribute to their stay(s) at the Y-PARC.

However, there was substantial variability in the extent and nature of the reported impacts. Two young people described profound and lasting improvements in their mental health. One of these young people reported that going to the Y-PARC probably saved her life at the time and that, longer term, her memories of experiences at the Y-PARC are an ongoing source of comfort.

*There isn’t a way for me to measure what they’ve done for me. Like I wouldn’t, I literally wouldn’t be alive if I didn’t have them. The very first time that I went to Y-PARC I was self-harming every single day and I was on, [laughs] my mum calls it “suicide watch” because she wouldn’t leave me alone … for more than like two seconds and it was horrible for her … And I remember going in there [Y-PARC] and it was like going to heaven [laughs]. Like they … were just angels to me and it’s always been a place of comfort. [Young person, Female #1]*

The family member interviewees of these young people similarly described how the service was critical in the young person’s recovery. Seven respondents reported noticing mild to moderate improvements that they could attribute to Y-PARC. Two young people reported little or no change and four reported improvements that they could not necessarily attribute to Y-PARC.

Four (of 5) family members spoke about the service as a genuine therapeutic alternative to staying in an inpatient mental health unit. One family member reported that her son did not benefit meaningfully from his stay at Y-PARC and attributed this to his short length of stay (approximately 1 week) and sedating effects of a psychotropic medication that he was prescribed upon commencement at the service.

All stakeholder respondents reported that Y-PARC is a worthwhile service that fills an important role in the service system, complementing community services and inpatient hospital services.

*It offers a great service for young people who are not quite ready to be out in the community on their own yet. I know that young people are admitted there after discharge from the mental health ward and if they didn’t have Y-PARC to go to, I think they would flounder. (Stakeholder #7)*

Specific impacts were most commonly reported in four areas:

- Coping and help seeking abilities.
- Personal growth and confidence.
- Understanding of mental health issues.
- Family dynamics and benefits for carers.

##### Coping and help seeking abilities

Most young people described Y-PARC making a positive contribution to what has been a journey of recovery or to a place of stability. Four out of five family members spoke about how Y-PARC helped to build the young person’s capacity to cope in the future.

*She’s not scared to ask for help as much anymore, as she was before. I think because she got the help in there that she now knows that if she does ask for help she’ll get it. (Family, #3)*

Just over one third of young people noted that, since their involvement with Y-PARC, they were now more comfortable and confident talking about their mental health issues, facing problems directly and asking for help.

*I think straight after Y-PARC it wasn’t … I was still in a pretty bad place. But I think that as I grew, matured, it kind of changed. So, I kind of stopped feeling depressed, I got out, had a job, socialised more, yeah, so that helped a lot. I mean like some days even now I do have you know, bad days, but I never ever think of you know, jumping in front of a train or – because that’s what I used to think about doing. [Young person, Female #4]*

##### Personal growth and confidence

Half of the young people spoke about having improved social skills and confidence. Several of the respondents described experiencing social anxiety. Being in the Y-PARC environment with people they did not know at first, but which was inclusive and supportive, helped develop the confidence and ability to push themselves further in socially challenging situations. Two family members spoke about how the young person benefited from exploring their independence in a safe environment and gained confidence because of their time at Y-PARC. Being around other young people was understood as being central to this.

*… you know, like when, when young people sit for too long on their own they become isolated. They don’t, you know, they’re not able to - they become socially inept and they’re not able to socially interact with others. So, to actually be taken out of, out of their safety zone, their home and whatever … She’s always known to go to these places but not as confidently as she does now on her own. She can self-initiate activities for herself … (Family, #2).*

##### Understanding of mental health issues

Y-PARC was described as having a role in mental health education by respondents in each group. Being in an immersive environment where mental health was the focus was emphasised by young people and families. Young people specifically spoke about improved awareness and ability to express their emotional state. Family members spoke about how young people benefited from the emphasis of staff on autonomy and self-care rather than solely on use of psychiatric medications to manage symptoms.

*I guess she had like a – rose-tinged glasses that somebody could just, sort of, take - you know, that medication was just going to make things better and things like that. So yeah, I think Y-PARC’s taught her that, no, it was an awful lot of hard work. (Family, #5)*

##### Family dynamics and benefits for carers

It appeared that the service also had a positive impact on family dynamics at least in the short term. Three young people spoke about how their family relationships had improved as a result of family work that took place at the Y-PARC. Two out of five family members reported that they personally benefited from opportunity for respite and their relationship with the young person improved as a result of time spent apart.

*Oh, it had a huge impact. Yeah, that was big for our family. Big, to have been able to finally voice issues that I’d had with my family. So, it was a big breakthrough that I did have. But yeah, in order for me to have done that, I did need safety and structure and support. Because I couldn’t do it on my own. There was no way. [Young person, Female #10]*

## Discussion

The evaluation identified that, over the three-year evaluation period, a total of 288 young people ages 16–25 years were resident at the Y-PARC service. Twice as many females accessed the service compared to males over the evaluation period (*n* = 194 to *n* = 94) and residents stayed for an average length of 19.6 days. Overall, this evaluation found that principles of recovery-oriented practice are embedded in the service, with young people, family and stakeholder respondents discussing how they valued and benefited from provision of person-centred care, promotion of autonomy and self-help, and time spent in an environment with other young people where they could focus on their mental health.

There was a high level of satisfaction reported by former Y-PARC residents and family members overall. Their accounts demonstrated that there is a strong emphasis on ensuring that residents feel safe and comfortable in the environment and it appeared that these elements were the building blocks of the therapeutic environment at Y-PARC. This is consistent with principles of ‘youth-friendly’ service design [43]. Being able to return for multiple stays at the service was an important service feature. This enabled young people to progress through several stages including stabilisation after a period of mental distress, gaining self-management skills, engagement and building of trusting relationships with staff over time. A flexible, self-paced, long term approach to care is also consistent with a recovery-oriented approach, which emphasises person-centred and user-led care [23].

Respondents also commonly reported that young people benefited from forming naturalistic, friendship-like relationships with staff and that the opportunity to do so was a key driver of engagement with other aspects of the service. It has been well established that the therapeutic relationship is an important facilitator of recovery for both young people and adults with aspects such as trust, respect and ‘caring’ being important to both [17, 18, 60]. However, the value placed on a friendship-like relational experience with staff among young people tends to be less prominent in studies with adults [20].

Findings from the current study suggested that provision of holistic, developmentally appropriate care was also critical, with one of the most widely discussed benefits being the opportunity to experience independent group living and to form new friendships with young people who shared similar experiences. This is consistent with recovery literature, which emphasises the importance of social connection and peer support, particularly in relation to skill development [21, 61].

There were some areas of challenge or tension identified in this evaluation. The data indicate that relationship-building and ‘the basics’ (e.g. independent living skills and social skills) were prioritised over the provision of structured formal interventions at the Y-PARC. There is evidence that some therapeutic techniques drawn from evidence-based models such as cognitive behaviour therapy may have been used by some staff members but none of our respondents across the four interview groups referred to consistent use of evidence-based psychosocial interventions. The delivery of evidence-based psychosocial interventions in residential youth settings is an under-developed area of the literature [62]. James et al. [39] argue that, while there is evidence to support use of several therapeutic interventions in youth residential settings, implementation is complex. Identified barriers include general receptivity among staff and clients, treatment factors and organisational/structural barriers [39]. Delivery of evidence-based psychosocial interventions was an area for continued investigation within this service with feedback from young people indicating that employment of a clinical psychologist, and structured group sessions may facilitate the delivery of more formalised interventions.

It was evident that family inclusive practice was a priority at the Y-PARC, with young people and family members giving numerous examples of how family and significant others (including children and friends) were included and welcomed by the service. The service also employed a specialist family engagement worker to support practice in this area. However, there was no evidence of implementation of evidence-based therapeutic family-focused interventions such as multi systemic therapy and functional family therapy [39, 62]. The difficulty of working therapeutically within the milieu of young people’s support networks including with family/carers is well-recognised in the mental health literature [63, 64], and was reflected in the data. Evaluation of an adult PARC service in Queensland found that involvement and engagement of informal carers was a challenge in that context; whereas, in this study, negotiating the appropriate level of involvement of family in care (e.g., decision making) was a more prominent issue. The issues associated with consent have been noted in both contexts [24, 28]. Moreover, the combined feedback from young people, family and staff reaffirmed that a continued emphasis on supporting staff to achieve family-inclusive practice was important.

One of the key innovative features of the Y-PARC and other emerging recovery-oriented residential environments is the provision of blended care (e.g. from clinical and non-clinical practitioners) through a partnership between multiple services. This arrangement was understood by Y-PARC staff and stakeholders as both a significant strength and a challenge. They described how, while negotiating different ‘languages’ was a challenge, they learned from one another and ultimately this arrangement contributed to a more holistic and seamless service.

The integration of psychiatric care into the model was a specific area of concern for many young people and parents. The psychiatric treatment model was experienced as inconsistent with the person-focused, recovery-oriented principles underpinning the broader Y-PARC model. There was also a perceived lack of continuity of care, with frequent changes in psychiatric personnel disrupting the capacity to build a therapeutic relationship. Young people who had a prior relationship with a private psychiatric practitioner preferred to maintain their existing relationship and many discussed issues associated with transfer of care.

When asked about service impacts, the majority of young people reported that they discerned improvements in their mental health due to their stay(s) at the Y-PARC and this was supported by family members. Stakeholders provided extensive feedback on the contribution that the Y-PARC makes to the system, with all reporting how it filled a ‘gap’ between community services and acute inpatient mental health hospital wards. There was some evidence that a stay at Y-PARC supplemented the need for hospital admission for some young people in this study. Family members and young people commonly contrasted the Y-PARC with hospital units, speaking about how the service focused on long term wellness rather than medication and risk management. While it is not possible to comment on long term outcomes for these young people, many spoke about improved resilience, better understanding of their mental health, an understanding of the importance of seeking help and stronger connections to therapeutic services moving forward – all of which are consistent with personal recovery [23, 26].

### Limitations

Although efforts were made to use a randomised sampling strategy, the number of qualitative interviews were small and still based primarily upon an opt-in strategy. The opt-in strategy is likely to bias the sample towards research participants who feel more strongly, either positively or negatively, about their experiences at the Y-PARC. The potential for biases in staff-reported practice is also acknowledged. In this respect it is important to note that staff accounts were only drawn upon in the current report when they were consistent with data collected from other sources. Finally, the evaluation was conducted very shortly after the first 3 years of establishment of the Y-PARC. It is well understood that community services undergo a period of rapid evolution and development following establishment. Follow-up research could be potentially conducted to investigate the progress of the service and to investigate changes that have been made following the first evaluation.

## Conclusion

Despite the abundance of attention to issues associated with mental ill health experienced among young people, the reality is that in Australia and elsewhere, young people and their families and communities experience a lack of availability of appropriate services and fragmented, inconsistent service delivery. This paper has outlined a service provision model that shows promise with this group, particularly in improving the range of availability of options across a spectrum of need.

## Data Availability

The datasets used and/or analysed during the current study are available from the corresponding author on reasonable request.

## Abbreviations

CBT: Cognitive behavioural therapy
DBT: Dialectical behavioural therapy
MHCSS: Mental Health Community Support Services
Y-PARC: Youth Prevention and Recovery Care service

## Footnotes

1 The Executive Summary of the report is publicly available via https://www.mindaustralia.org.au/sites/default/files/Evaluation_of_the_Frankston_YPARC_executive_summary.pdf.

2 Youth co-researchers were recruited from a Youth Research Council established by Orygen, Australia’s National Centre of Excellence in Youth Mental Health.
